# Hyperbaric Oxygen Post Established Stroke

**DOI:** 10.7759/cureus.63395

**Published:** 2024-06-28

**Authors:** David W Harrison, Penny M Brasher, Janice J Eng, Devin Harris, Alison M Hoens, Afshin Khazei, Jennifer K Yao, Riyad B Abu-Laban

**Affiliations:** 1 Emergency Medicine/Hyperbaric Medicine, Vancouver General Hospital/University of British Columbia, Vancouver, CAN; 2 Centre for Clinical Epidemiology and Evaluation, Vancouver General Hospital/University of British Columbia, Vancouver, CAN; 3 Physical Therapy, GF Strong Rehabilitation Centre/University of British Columbia, Vancouver, CAN; 4 Emergency Medicine, Kelowna General Hospital/University of British Columbia, Kelowna, CAN; 5 School of Population & Public Health, Centre for Clinical Epidemiology & Evaluation, Arthritis Research Canada, Centre for Health Evaluation & Outcomes Sciences, University of British Columbia, Vancouver, CAN; 6 Physical Medicine and Rehabilitation, GF Strong Rehabilitation Centre/University of British Columbia, Vancouver, CAN; 7 Emergency Medicine, University of British Columbia, Vancouver, CAN

**Keywords:** hyperbaric, ischemic stroke, stroke rehabilitation, stroke recovery, rehabilitation, hyperbaric oxygen, oxygen, stroke

## Abstract

Background and purpose: Hyperbaric oxygen therapy (HBOT) has been reported to improve neurological function in the chronic phase of stroke in a single trial having significant limitations, including a lack of a sham control.

Methods: We conducted a single-center, parallel-group, randomized trial to determine the effectiveness of HBOT compared with a sham control in adults who were 6 to 36 months post-ischemic stroke. The treatment group received 40 sessions of HBOT at the Vancouver General Hospital Hyperbaric Unit. The control group received 40 sessions of sham treatment designed to replicate an HBOT experience.

Due to recruitment challenges and timeline/feasibility tracking by the research team, the control arm was altered after 20 months to a waitlist in the hope of increasing participation. In the second phase, participants were randomized to receive HBOT immediately or following an eight-week observation period.

The primary outcome was the post-treatment Stroke Impact Scale-16 (SIS-16). Secondary outcomes included the National Institute of Health Stroke Scale, Berg Balance Test, Digit Symbol Substitution Test, 5-Metre Walk Test, 6-Minute Walk Test, Grip Strength, Montreal Cognitive Assessment, Box/Block Test, and Center for Epidemiological Studies - Depression and Short Form-36. Based on detecting a clinically important between-group difference of 10 on the SIS-16 score, our target sample size was 68 participants per arm.

Results: From January 5, 2016 to October 9, 2018, 34 participants were enrolled in the trial, 27 during the first phase and seven in the second phase. The study was stopped after 36 months, and prior to meeting the sample size target, due to low recruitment. At the end of treatment, the difference in the SIS-16 between groups was 5^.^5 (95% CI: 1^.^3 to 9^.^7, p = 0^.^01) in favor of the sham group.

Conclusions: Our results exclude a clinically important benefit of HBOT on the primary outcome of the SIS-16. These findings do not support the use of HBOT in chronic stroke survivors.

## Introduction

Stroke is a major cause of mortality and long-term disability. The lifetime risk of stroke is approximately 25% [1]. Over half of stroke victims have some disability at one year [2]. Furthermore, stroke accounts for over 4% of all direct healthcare costs in high-income countries [3]. The estimated annual stroke-related cost of healthcare, lost wages, and decreased productivity in Canada is approximately $3.6 billion [4].

Most of the neurological improvement among stroke survivors occurs in the first 30 to 90 days [5]. Further modest functional improvements are possible after this period with interventions from an interdisciplinary rehabilitation team, but major disability often persists [2]. Hyperbaric oxygen therapy (HBOT) has been advocated and used by some healthcare providers as a therapy to improve disability post-stroke. HBOT involves the administration of inhaled 100% oxygen at increased ambient pressure inside a closed vessel, producing greatly elevated arterial and tissue oxygen tensions, and a wide variety of physiological effects at the cellular and sub-cellular levels [6,7].

HBOT has been studied as a treatment in the acute/subacute phase and chronic (3-6 months post-stroke) phase of stroke. A 2014 Cochrane systematic review of HBOT for acute ischemic stroke found that "there was no good evidence to show that HBOT improves clinical outcomes when applied during acute presentation of ischemic stroke", although the possibility of clinical benefit had not been excluded [8].

The use of HBOT in chronic stroke has been less studied. A PubMed search for randomized controlled trials of HBOT in chronic stroke (Hyperbaric Oxygenation/ AND (Stroke Rehabilitation/ OR Stroke/) AND randomized controlled trial.pt.) published between 1960 and 2024 identified one publication. There were significant limitations with the trial: it used a waitlist control, which can lead to overestimates of treatment effects [9,10], and the validity of the primary outcome, NIHSS, is uncertain in chronic stroke [11].

In an attempt to more definitively evaluate the use of HBOT as a treatment in the chronic phase of ischemic stroke, we designed a randomized clinical trial comparing the effect of sham HBOT and true HBOT on the neurological status of participants who were between six months and three years post-stroke. We hypothesized that a course of 40 treatments of HBOT would improve neurological function, as reflected in Stroke Impact Scale-16 (SIS-16) scores.

## Materials and methods

Study design and participants

We designed a single-center, randomized trial comparing HBOT with a sham control. The trial was conducted at Vancouver General Hospital (VGH), Vancouver, Canada. VGH is a tertiary care teaching hospital associated with the University of British Columbia. The VGH Hyperbaric Unit provides approximately 3,700 elective and emergency treatments to 175 patients annually for the 15 conditions approved for HBOT by the Undersea and Hyperbaric Medical Society (UHMS) and Health Canada. Adults (age 19-85 years) with a confirmed diagnosis of one ischemic stroke involving the cerebral hemispheres or cerebellum within the previous 6 to 36 months, and who had a score of less than 70 on the SIS-16 stroke scale were eligible.

We chose the time frame of 6 to 36 months to be consistent with the only other study examining HBOT in the chronic phase of stroke [9]. The six-month delay in starting treatment is beyond the time frame where the majority of neurological recovery occurs post stroke. It was also selected so as to not interfere with other effective occupational therapy and physiotherapy.

Participants had to be capable of transferring into the hyperbaric chamber unaided or with a one-person assist, and to sit in the chamber with the assistance of a waist and chest strap, if needed, for 120 minutes. Exclusion criteria included standard contraindications to HBOT, current participation in other stroke studies, a previous stroke more than 36 months prior to recruitment, and multiple ischemic strokes within the 6-36 months prior to recruitment. The University of British Columbia Clinical Research Ethics Board approved the study (approval H15-00766) and it was registered at clinicaltrials.gov prior to commencing enrollment (NCT02582502). All participants provided written informed consent.

Potential participants were identified by a wide variety of mechanisms, chart reviews of all patients discharged from local hospitals with a diagnosis of ischemic stroke, advertising through conventional media outlets, social media, posters, stroke support and recovery groups, and through direct communication with clinics and care providers who were potential referral sources. These screening procedures identified 3,883 individuals with a possible diagnosis of ischemic stroke. Potential participants identified from hospital discharge data were sent a letter and followed up with a phone call. Clinicians also identified potential participants during clinic visits, and if the patient expressed an interest they were contacted by phone. During the phone call, individuals were invited to provide written permission to review their medical records to determine eligibility for the study. If potential participants met eligibility criteria, they were then contacted by telephone to complete an SIS-16 questionnaire. A score of less than 70 out of a potential 100 on the SIS-16 made them eligible for an in-person assessment to confirm eligibility and to provide written consent. We chose a score of less than 70 as a selection criterion in order to ensure that participants had at least moderate deficits, sufficient to justify a prolonged and expensive treatment regimen and to have the potential for a measurable and clinically significant improvement, which we defined as an increase of 10 on the SIS-16.

Randomization and masking

Participants were randomized after eligibility was established and baseline data collected. The baseline assessment was conducted in person by a contracted staff member of the Vancouver Coastal Health Research Institute Clinical Research Unit. The completed assessment package was immediately reviewed by the research coordinator to verify eligibility. If the participant remained eligible, the research coordinator accessed the randomization list for the next allocation. The research nurse then contacted the participant with the date and time of their first treatment. They were assigned (1:1) to either the treatment or sham group. The randomization list was created by the research coordinator using the online tool, Sealed Envelope. A total of 168 allocations was created using permuted blocks of 6. The list was kept in a password-protected Excel file and only the research coordinator had access to the file.

Study participants and outcome assessors were blinded to the participant’s treatment. Study staff were not blinded, so as to ensure the safety of research staff and participants while inside the hyperbaric chamber. Participants were asked after the fifth treatment day to indicate the group to which they believed they had been randomized. Participants were not eligible to be informed of their randomization assignment until all study data had been collected for all study participants.

Procedures 

The treatment group received 40 sessions of HBOT for 90 minutes at 2 atmospheres absolute (ATA), equivalent to 33 feet of seawater or 120.9 kPa, in a multi-seat chamber. Treatments were administered 5 days a week (not including statutory holidays and Hyperbaric Unit closures). The control group received 40 sessions of sham HBOT. The sham treatment was a sophisticated protocol designed to simulate the actual HBOT as closely as possible. Participants were prepared and placed in the chamber in the same manner as participants receiving HBOT. The chamber was pressurized with air to five feet of seawater (1.15 ATA) to provide a mild sensation of pressure on the tympanic membranes of the ears. During this sham pressurization the supply and exhaust of air into the chamber was adjusted to simulate the sounds normally heard during compression. The temperature of the chamber was also adjusted to simulate warming with pressurization and cooling with depressurization. Pressure gauges were not visible to participants inside the chamber. After 10 minutes of simulated pressurization chamber, occupants were placed on hoods for simulated alternating oxygen and air breaks in a schedule to simulate HBOT. After the first 10 minutes, the chamber was returned to a depth of one foot of seawater (1.03 ATA). Treatment and control groups were kept separate at all times in order to prevent any sharing or comparison of experiences between treatments.

In response to low enrolment numbers, the methodology was altered after 20 months of study operation to a waiting list trial. Participants from this point forward were randomized to receive either immediate HBOT, or to receive HBOT following an eight-week waiting period, followed by re-evaluation. Both groups were similarly assessed before any intervention by an assessor who was blinded to their allocation.

Outcomes

Participants were followed up for one year. The primary outcome measure was the Stroke Impact Scale-16 (SIS-16) at the end of treatment. Participants were assessed immediately after finishing HBOT, and at two months, six months, and 12 months post treatment. The SIS-16 evaluates the impact of stroke on physical functioning, including mobility and activities of daily living [12]. Scores range from 0 to 100 with higher scores reflecting better function.

Secondary outcomes measured physical impairments and function, and quality of life using the following scales: National Institute of Health Stroke Scale [13]; Grip strength (Hydraulic Hand Dynamometer, Model SH5001, Saehan Cooperation) [14]; 5-Meter Walk test [15]; 6-Minute Walk test [15]; Berg Balance Scale [16]; Box and Block Test [17]; Montreal Cognitive Assessment [18]; Digit Symbol Substitution Test [19]; Center for Epidemiologic Studies Depression Scale [20]; 36-Item Short Form Survey [21].

Statistical analysis

The primary outcome was SIS-16 at the end of treatment, with a difference of 10 or greater deemed a priori to be clinically significant, in keeping with prior research [22]. Assuming a Type 1 error of 5% (2-sided), a standard deviation of 20, and a correlation of 0.25 between the baseline and end of treatment scores, we estimated that a sample size of 68 per group would have 85% power to detect a difference of this magnitude or greater. The sample size was based on a comparison of two groups, thus the change from a sham control to waitlist control did not impact the target sample size.

Baseline characteristics were described by the treatment group. Continuous data were summarized as mean (with standard deviation) and categorical data as frequencies and proportions. Analysis was by randomized group irrespective of whether the patients adhered to their treatment assignment. Between-group differences were estimated from linear mixed models (LMM) using restricted maximum likelihood and constraining the baseline values to be equal [23]. This method is essentially equivalent to an analysis of covariance.

Group and time were included in the model as fixed effects, and the participant was included as a random effect. Missing values were not imputed as the LMM provides statistically efficient, unbiased estimates when data is missing at random [24]. Secondary outcomes were assessed simultaneously using O'Brien's global statistical test. For secondary outcomes where higher scores represent worse function, the values were negated so that all scores were in the same direction for the global test. Estimates of between-group differences were determined as for the primary outcome. All analyses were performed using Stata version 16.1 (Statacorp LLC, College Station, Texas) and R version 3.6.1 (R Foundation for Statistical Computing, Vienna, Austria).

## Results

Thirty-four participants were enrolled between January 5, 2016 and October 9, 2018; 27 were in the first phase and 7 in the waiting list phase. The study was stopped after 36 months, and before reaching the sample size target of 136 participants because of continued enrolment challenges. Participants were mild to moderate in stroke severity, with a mean SIS-16 of 57.1. SIS-16 scores of 32, 63, and 76 have been shown to correspond to moderately severe, moderate, and slight disability respectively on the modified Rankin Scale [25].

**Figure 1:**
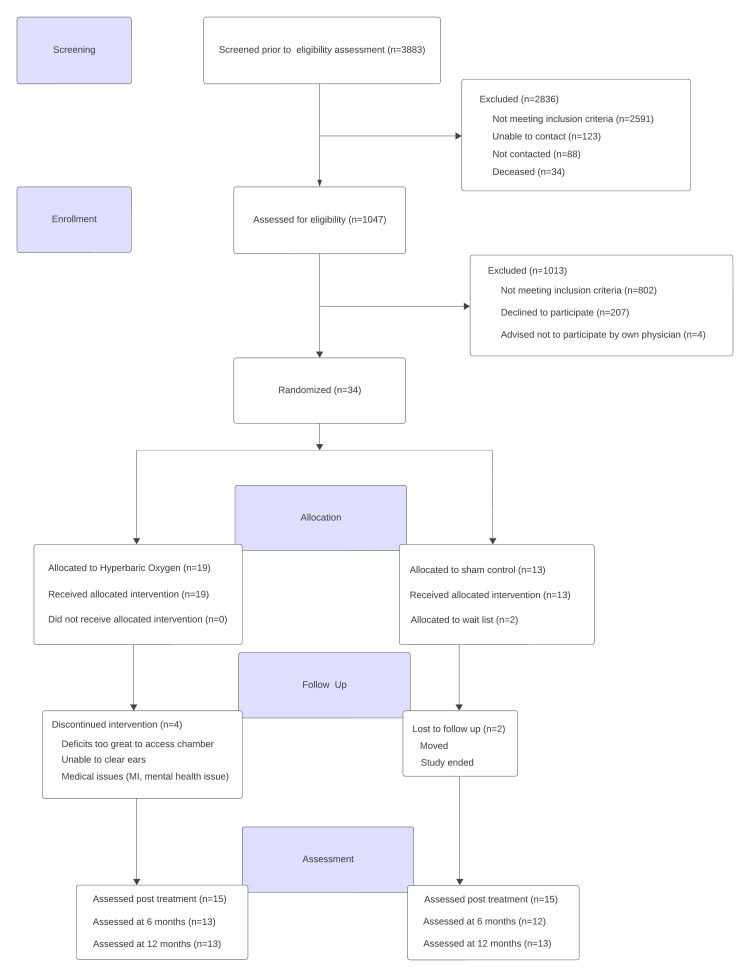
Consort Flow Diagram

Figure 1 shows the flow of participants. Between October 2015 and October 2018, 3,883 individuals were assessed for eligibility. The final follow-up visit occurred in January 2020.

**Figure 2:**
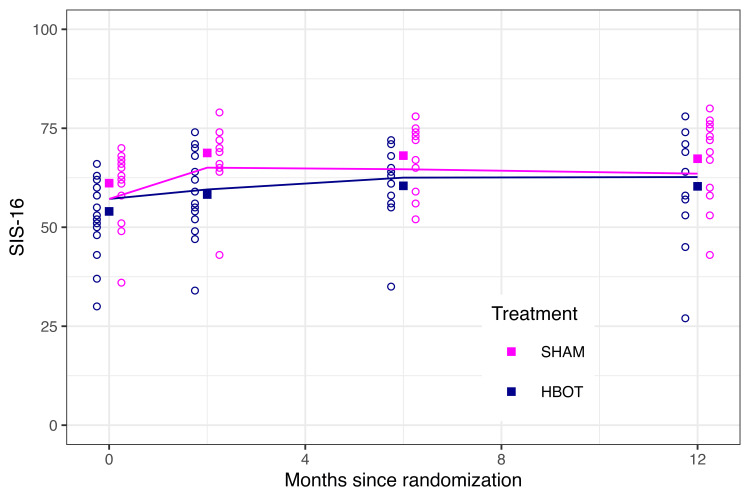
SIS-16 Scores for the Treatment Group SIS-16, Stroke Impact Scale-16; HBOT, Hyperbaric Oxygen Therapy

At the end of treatment, the difference in SIS-16 between groups was 5.5 (95% CI: 1.3 to 9.7, p = 0.01) in favor of the sham group. The difference found in the end of treatment SIS-16 was not sustained over time (Figure 2). The sham group was therefore statistically better than the HBOT group post-intervention. However, the group difference of 5.5 did not meet our a priori 10-point threshold for a clinically significant difference.

Patient characteristics at baseline are shown in Table 1. The average scores at baseline for the primary and secondary outcomes are provided in Table 2.

**Table 1:** Patient Characteristics at Baseline HBOT, Hyperbaric Oxygen Therapy

	HBOT (n = 19)	Sham (n = 15)
Age in Years: Mean (SD)	64.6 (12.7)	60.4 (10.4)
Males/Females: Number	13 / 6	14 /1
Years of Education: Mean (SD)	14.1 (4.1)	14.8 (4.4)
Smoker: Number	0	0
Years Since Stroke: Mean (SD)	1.4 (0.7)	1.3 (0.7)

**Table 2:** Outcome Measures at Baseline Results are expressed as mean and standard deviation (SD) unless otherwise indicated. SIS-16, Stroke Impact Scale; NIHSS, National Institute of Health Stroke Scale; MoCA, Montreal Cognitive Assessment; MoCA < 26, Montreal Cognitive Assessment < 26; DSST, Digit Symbol Substitution Test; CES-D, Center for Epidemiologic Studies Depression Scale; CES-D >16,  Center for Epidemiologic Studies Depression Scale >16; SF-36 PCS, Short Form Health Survey Physical Component Score; SF-36 MCS, Short Form Health Survey Mental Component Score; BBT, Box and Block Test; HBOT, Hyperbaric Oxygen Therapy

	HBOT (n = 19)	SHAM (n = 15)
SIS-16	54.0 (9.6)	61.1 (9.3)
NIHSS	6.9 (3.7)	5.5 (4.5)
MoCA	20.2 (7.9)	21.3 (5.8)
MoCA < 26 (number)	12	12
Berg balance test	43.8 (15.1)	49.2 (8.4)
Digit substitution test	16.9 (10.6)	19.3 (9.6)
6-minute walk test	212.2 (155.4)	242.3 (128.3)
5-metre walk test	13.2 (24.3) [n=16]	7.2 (6.4) [n=13]
CES-D	18.5 (9.3)	16.1 (9.9)
CES-D >/= 16 (number)	11	9
SF-36 PCS	31.1 (6.1)	34.5 (8.1)
SF-36 MCS	54.7 (9.8)	51.8 (18.8)
BBT	7.8 (13.4)	11.9 (16.4)
Grip strength	7.6 (10.8)	10.6 (12.3)

The global test for the secondary outcomes yielded p=0.69, and results for each outcome are provided in Table 3. For comparison to the results of the prior randomized trial that concluded a benefit exists [6], the estimated between-group difference (HBOT - sham) for the NIHSS post-treatment scores was -0.78 (95% CI: - 2.23 to 0.66).

**Table 3:** Results for Secondary Endpoints NIHSS, National Institute of Health Stroke Scale; CES-D, Center for Epidemiologic Studies Depression Scale; SF-36 PCS, Short Form Health Survey Physical Component Score; SF-36 MCS, Short Form Health Survey Mental Component Score; BBT, Box and Block Test; HBOT, Hyperbaric Oxygen Therapy

	LS mean (95% CI) difference post-treatment (HBOT – SHAM)
NIHSS	-0.8 (-2.2, 0.7)
MoCA	1.2 (-0.7, 3.0)
Berg balance test	0.4 (-2.2, 3.0)
Digit substitution test	0.0 (-3.4, 3.3)
6-minute walk test	-3.0 (-40.2, 34.3)
5-metre walk	0.2 (-0.8, 1.1)
CESD	-2.3 (-8.9, 4.3)
SF-36 PCS	-0.7 (-6.3, 4.9)
SF-36 MCS	-3.2 (-9.7, 3.4)
BBT	-3.2 (-7.3, 0.9)
Grip strength	-2.6 (-6.9, 1.6)

Sham effectiveness

Twenty-five patients in the first phase were assessed for blinding. Participants were unable to tell if they received actual HBOT or sham treatments. Five of 12 sham and four of 13 HBOT patients thought they were receiving active treatment.

Adverse events

We identified two adverse events during the study, neither of which were felt to be directly related to the sham or treatment protocols. One participant undergoing HBOT experienced ischemic chest pain while in the chamber. This individual had been experiencing events outside the chamber for several days but did not report his symptoms to research staff until they occurred during treatment. The individual was safely removed from the chamber and sent to the emergency department for assessment. He underwent a triple vessel coronary artery bypass graft and was subsequently withdrawn from the study. A second participant who had been found to be very unsteady on the Berg Balance Test fell during an initial assessment, while attempting to spontaneously stand independently. Fortunately, only minor contusions were sustained.

## Discussion

Stroke is not among the 15 indications for HBOT approved by the UHMS [7]. Despite this and a lack of compelling evidence of benefit, it is provided as a treatment for chronic stroke in many private, for-profit, user-pay hyperbaric facilities in North America and elsewhere.

The only previous study of HBOT in chronic stroke by Efrati et al. reported a reduction in disability when patients (N = 59) were treated with HBOT 6 to 36 months after ischemic or hemorrhagic stroke [9]. This was a randomized trial that provided 90 minutes of HBOT at 2 atmospheres absolute (ATA) for 40 sessions [9]. Fifty-nine patients who had suffered an ischemic or hemorrhagic stroke 6 to 36 months previously were included, and participants receiving HBOT were reported to have statistically significant improvement in neurological function, as reflected by the National Institute of Health Stroke Scale (NIHSS), activities of daily living (ADL), and quality of life (QoL) scores. The average improvement in the NIHSS score was modest (2.75 points) and sustained. This study was described by the authors as a randomized crossover trial, although it failed to meet the prerequisite for a cross-over trial since the intervention had a sustained effect, precluding a washout phase. Details about the type and location of the stroke (thromboembolic, lacunar infarct, etc.) were not provided. All patients were initially assessed with SPECT scanning, NIHSS, ADL, and quality of life scores. Subjects were then randomized to one of two arms. The initial treatment arm received HBOT for 90 minutes at 2 ATA for 40 sessions, followed by a repeat of their initial assessment [9]. The subjects in the crossover arm were observed during the initial period during which the other subjects were treated. They were then reassessed for any changes. Following their reassessment, they received HBOT for 90 minutes at 2 ATA for 40 sessions and a final assessment. Both groups showed statistically significant improvement in neurological function, ADL, and quality of life scores following HBOT but not after the control observation period. SPECT scans correlated with clinical improvements in both groups [9].

Although the Efrati study is widely cited as justification for treating chronic stroke with HBOT, it had significant limitations. The primary neurological outcome in the study was the NIHSS [9]. However, this score was developed as a measure of acute stroke severity used to predict the outcome and guide early intervention. The utility of the NIHSS in chronic stroke recovery studies is unclear [11]. The mean NIHSS scores for the treatment and control groups before HBOT were 8.53 +/- 3.62 and 8.34 +/- 4.25 respectively. Mean scores after HBOT were 5.52 +/- 3.59 and 5.85 +/- 3.44. Thus, the mean change in the NIHSS after HBOT for all subjects was 2.75 [9]. Although the authors reported this as a statistically significant change, the clinical significance of this magnitude of change on the NIHSS is unclear and would depend on the nature of the disability. These issues were not clarified by the authors [9]. Efrati used an unidentified ADL score [9]. The mean change from before to after HBOT was 3.48. This change was reported as statistically significant, but sufficient clinical correlates were not provided [9]. The QoL score used by Efrati was the EQ-5D [9]. The format in which the results were reported in the study was somewhat unconventional, making interpretation difficult. In summary, a number of issues related to this study require clarification. Our randomized clinical trial was carefully designed to, wherever possible, avoid these limitations.

Outcome measures

The assessment of neurological function after stroke is a complex task; hence, outcome measures must be known to validly reflect patient activity and participation. We chose the SIS-16 as our primary outcome because it is a well validated, multi-dimensional measure of activity and participation post-stroke [12]. The SIS-16 reflects the impact of stroke on physical functioning and contrasts with the NIHSS, which has only been validated as a measure of acute neurological impairment [11]. While we collected data on the NIHSS so that we could compare our results to those of previous investigators who used the NIHSS as their primary outcome [9], we do not feel the NIHSS is an appropriate primary outcome for a study of chronic stroke. We also included a variety of secondary outcomes often used in assessing stroke recovery, both for hypothesis generating purposes and to better understand any identified impact on our primary outcome of SIS-16. 

Functional recovery from stroke can continue up to three years post-stroke, depending on the remaining integrity of neuronal pathways [26]. Hence, a robust study of post-stroke recovery must distinguish between expected functional recovery over time and improvements attributable to any specific intervention. Because of this, having an effective sham in an HBOT study of stroke to minimize any placebo effect, particularly in a situation of self-reported outcomes, is of great importance.

Sham control

Designing an effective sham for HBOT is a challenge when designing a randomized clinical trial of HBOT. A recent review of double-blind trials in hyperbaric medicine identified 42 studies in which various sham hyperbaric treatments were employed [27]. They classified the sham treatments into three broad categories: (1) use of a lower pressure than that of the hyperbaric oxygen group, while breathing 21% oxygen; (2) use of the same pressure as the hyperbaric oxygen group, while breathing an adjusted percentage of oxygen; and (3) use of the same pressure as the hyperbaric oxygen group, while breathing 21% oxygen [27]. Our sham treatment of breathing air at a pressure of 38 feet of seawater for 10 minutes (1.15 ATA) and at 34 feet of seawater (1.03 ATA) for 90 minutes is the equivalent of breathing 24% oxygen and 21.6% at the surface and was similar to others described in their review [27]. The authors concluded that this type of sham using a lower pressure than the hyperbaric oxygen group while breathing 21% oxygen best matches the inertness of the placebo [27].

Ours is the first randomized clinical trial using sham HBOT in the study of chronic stroke. Since our study used both externally administered and self-reported measures, we believe employing a reliable sham HBOT was essential to the appropriate interpretation of our findings. We assessed the validity of our sham protocol and found that participants were not able to distinguish between actual HBOT and sham. The finding underscores the validity of our sham protocol and reinforces the effectiveness of employing sham placebos in any future studies of HBOT in stroke.

Findings

Our study did not demonstrate any clinically significant improvement in physical or neurological functioning after treatment with HBOT in chronic post-stroke participants. In fact, our results suggested that the HBOT treated group had worse functional outcomes immediately following their eight-week course of HBOT, compared to sham controls. While this could be a spurious result, it is also possible that it was a side effect of HBOT.

Fatigue is a common side effect of high oxygen concentrations experienced during HBOT [7,28]. Our initial post treatment assessment was done as soon as possible after completing HBOT, with 13 of 39 assessments (33%) carried out on the same day or first day after finishing treatment, and 21 of 39 (54%) within three days. Fatigue experienced by the HBOT treatment group may have caused a reduction in other rehabilitation activities during the course of their HBOT, resulting in reduced SIS-16 scores. Residual fatigue may have also been present at the time of the first assessment, resulting in a direct effect of reduced scores.

Limitations

We did experience some unexpected challenges in conducting the study, leading to certain limitations. We undertook a comprehensive strategy to reach participants and included all avenues recommended by others in the field of stroke rehabilitation [29]. Despite this, we still encountered significant recruitment challenges, with the large time commitment being one of the most common reasons for declining participation [29]. In surveying candidates who declined enrollment, we discovered that uncertainty about receiving sham vs actual HBOT, the time commitment required of the study, with disruption of other rehabilitation activities and the logistical challenges of travel to and from the hyperbaric unit were significant factors. The lack of recruitment, which jeopardized the study viability, prompted us to add the waiting list phase of the study. Unfortunately, this did not improve recruitment, suggesting that the sham was not a major factor in our recruitment challenges. Our experience suggests that future investigators should anticipate and have a strategy for dealing with similar issues.

In a study of this nature that fails to meet its original projected sample size requirement, the appropriate approach is not to focus on the original power calculation but rather on the actual point estimates and their 95% confidence intervals. Although target recruitment was not reached, our results exclude a clinically important benefit of HBOT on the primary outcome of SIS-16. It is possible our results were influenced by the slightly younger age and baseline less severe NIHSS of our sham group. However, our study failed to reproduce the previously reported benefits of HBOT as measured by the NIHSS [9]. Our study also demonstrated the effectiveness and safety of the rigorously applied sham HBOT treatment protocol we designed. We believe that any future studies should employ a similar sham group. Our results do not support the use of HBOT in chronic stroke survivors and raise significant concerns regarding private enterprises offering HBOT to stroke patients, typically at great cost. 

## Conclusions

We designed a randomized clinical trial comparing the effect of sham HBOT and true HBOT on the neurological status of participants who were between six months and three years post-ischemic stroke. Our results do not concur with the previously published study which reported a modest benefit of HBOT in chronic stroke. In contrast, we found a statistically significant benefit in favor of sham HBOT that did not meet our a priori 10-point threshold for a clinically significant difference.

The available evidence does not support the use of HBOT in chronic stroke, except in the context of well-designed randomized trials. Future studies should include a sham control, which was demonstrated to be effective in our study. Pending further evidence supporting its effectiveness in treating chronic stroke HBOT should not be used as a therapeutic modality, given its significant cost and lack of proven benefit.
